# Leisure-Time Physical Activity Participation Trends 2014–2018: A Cross-Sectional Study in Poland

**DOI:** 10.3390/ijerph17010208

**Published:** 2019-12-27

**Authors:** Elżbieta Biernat, Monika Piątkowska

**Affiliations:** 1 Collegium of World Economy, Warsaw School of Economics, Al. Niepodległości 162, 02-554 Warsaw, Poland; 2 Faculty of Physical Education, Josef Pilsudski University of Physical Education in Warsaw, 34 Marymoncka, 00-968 Warsaw, Poland; monika.piatkowska@awf.edu.pl

**Keywords:** leisure-time, physical activity, time-trend perspective, determinants, health recommendations, Poland

## Abstract

Objective, the aim is an analysis of sociodemographic factors that had an essential relation with undertaking leisure-time physical activity—LTPA (with particular emphasis on World Health Organization (WHO) health recommendations) by adult Poles from a time-trend perspective. Methods, the paper is based on data retrieved from five large-scale surveys carried out on the representative samples of Poles aged 15–69 in 2014–2018 (*n* = 7347). In each wave, the Polish long version of the International Physical Activity Questionnaire was used. Results, the participation of Poles in LTPA constantly increases (*p* < 0.001) and the percentage of people meeting the dose of PA required for health recommendations is growing (on average, met by 43.9% of Polish men and 43.5% of women), which indicates a trend of behaviour of citizens of this country. The probability of realisation of WHO norms was determined by sex, age, place of residence and occupational status. Conclusion: it seems reasonable to develop Polish recommendations and guidelines for specific groups, including females and males, those who live in the villages and in the towns, different age groups: children/adolescents, adults (aged 50 to 59—most vulnerable to physical inactivity), the elderly, and various professional groups (especially farmers and physical workers).

## 1. Introduction

In the modern world, physical activity (PA) ceases to be an individual and personal matter and is becoming both an essential social issue and an element of policy. Its unique importance is perceived by public health institutions [1,2] and governments all over the world, and has established the increase of PA as a priority activity [3,4] (especially due to increasing concerns over the negative health effects of a deficit of activity) [2,5]. In the second half of last year in Europe, as a result of these activities (undertaken at regional, national, and individual levels [1]), changes in the consciousness and lifestyle of people, including a significant growth in the number of people participating in sport, recreation and tourism, were noted [6]. In our current reality, these activities are, generally, the only possibility of levelling up disproportions in the absence of activity [7,8]. However, at the same time, about 60% of Europeans do not participate in sport, or do so very rarely [9]. This is an essential factor in the risk of non-communicable chronic diseases (NCDs) [1,10].

Changes in behaviour in modern societies require the discovery and understanding of current factors that condition leisure-time physical activity (LTPA). In Europe, since the end of the 1970s, new phenomena have been noticed which are closely connected with the undertaking or avoidance of PA [11]. For example, an increase in the share of women in sport is observed, at an equal level to men [12]. Danish and Dutch studies show that women are even more active than men [11,13,14]. In general, younger generations are still more physically active than older ones [15,16]. However, during the last three decades, significant growth in participation in sport by older people has been noticed [9,16,17]. Lera-López and Rapún-Gárate [14] claim that this is a result of (1) the greater resources of this age group, (2) an increase of educational status (along with knowledge and awareness of the need of undertaking PA [18]. Scheerder and Vos [11] state that the increase in the level of education in modern societies has resulted in the growth of participation in sport for all educational groups. One can say that current differences between the groups mentioned above are becoming blurred, although the fact that educational status determines participation in sport is still visible [11]. A positive relationship between social and economic status (mostly educational) and LTPA is confirmed by other scientists [19,20,21]. However, Hallmann and Breuer [17] point out the fact that an increase in education level (and often related professional advance) can lead to larger time limitations. Similar results are presented by other transnational studies [13,22].

It appears crucial to detect lifestyle behaviour trends at an early stage and to identify their determinants in order to develop effective interventions [23]. Considering this type of knowledge in modern strategies which focus on increasing the level of PA is an inevitable element of the success [24]. Thus, the aim of this paper is to analyse sociodemographic factors which have had an essential impact on Polish adults undertaking LTPA from a time-trend perspective. The study considers three key areas, i.e., (1) evaluation of the overall PA level of Poles; (2) evaluation of World Health Organization (WHO) recommendations concerning the amount of PA optimal for health; (3) evaluation of the actual influence of selected sociodemographic factors on pro-health PA level among the population under study. Nationwide cross-sectional research was realised annually (2014, 2015, 2016, 2017, 2018) in a random group of over 1000 people. To the best of our knowledge, a study of such type using Polish data has been conducted for the first time.

## 2. Materials and Methods

### 2.1. Data Collection

The paper is based on data retrieved from five large-scale surveys used to collect information on the PA of Polish society. All surveys were conducted by order of the Ministry of Sport and Tourism of the Republic of Poland. The data were gathered on representative samples of Poles aged 15–69 in 2014 (*n* = 1019), 2015 (*n* = 1020), 2016 (*n* = 2118), 2017 (*n* = 2131) and 2018 (*n* = 1059). In each wave, the sample was random-quote and selected from the sampling frame of the National Official Register of the Territorial Division of the Country (TERYT). The sampling procedure included three stages: territorial stratification, drawing addresses, and allocation of demographic characteristics. Computer-assisted personal interviews (CAPI) were conducted by trained and supervised pollsters. The ethics committee of the Polish Academy of Sciences approved the study (approval nr. KEwN/60/2014) in accordance with the Declaration of Helsinki (2004). Participation was voluntary and confidential, and informed consent was obtained from participants before completing the survey.

In each wave the same standardized questionnaire—the Polish long version of the International Physical Activity Questionnaire-Long Form [25] (IPAQ-LF)—was used. IPAQ-LF provides information about the frequency, duration, and intensity of activities during the previous seven days. The questionnaire assesses four domains in which PA is performed: leisure time, domestic, occupational, and transportation. In this study, only questions on LTPA, including vigorous PA (VPA), moderate PA (MPA) and walking, were analysed. LTPA refers to exercise, sports or recreation which is not related to regular work, housework, or transport activities [26]. The minimum duration of a single PA is set at 10 min.

Due to the consistency in data collection, questionnaires and sampling, the repeated cross-sectional design of this research allows time-trend analysis to be incorporated [27].

### 2.2. Participants

The sample comprised 7347 Poles (3551 males and 3796 females) aged 15–69 years old. Sex, age (15–29 years, 30–39 years, 40–49 years, 50–59 years, 60–69 years), level of education (primary, basic vocational, secondary and higher), place of residence (village, towns up to 500,000 inhabitants, towns with 500,000 inhabitants) and occupational status (white-collar workers, physical workers, farmers, housewives, retirees/pensioners, students, unemployed) were considered as sociodemographic indicators for descriptive analysis. Table 1 provides descriptive statistics for the five subsamples.

### 2.3. Data Analysis

Data were prepared and analysed according to IPAQ guidelines for data processing and analysis [28]. From the initial sample (*n* = 7450) cases with missing data (*n* = 87) and ‘unreasonably high’ values (reports of activity in excess of 16-h/day considered implausible) (*n* = 16) were from the study (*n* = 103). A final sample (*n* = 7347) was used for further analysis.

The IPAQ provides total activity scores expressed as MET/min (MET corresponds to the consumption of O_2_ during resting and =3.5 mL O_2_/kg of body mass per minute.) a week and constituted the sum of weekly energy expenditure on VPA, MPA and walking. The weekly energy expenditure for each of three activities was calculated by multiplying the MET value assigned to it (VPA—8 MET, MPA—4 MET, and walking—3.3 MET) by the number of days of it was performed during a week, where MET corresponds to O_2_ consumption during the rest and equals to 3.5 mL O_2_/kg of the body mass per minute. The cut-off points of LTPA doses according to WHO recommendations were adopted [1]. Meeting the WHO recommendations on PA for health meant taking up at least 150 min of moderate-intensity, or 75 min of vigorous-intensity, PA per week, or any equivalent combination of the two.

### 2.4. Satitistical Analysis

The data collected by the questionnaires were analysed using IBM^®^ SPSS^®^ Statistics ver. 22 (IBM Corporation, Armonk, NY, USA). A descriptive analysis was performed to explore the sample characteristics (frequencies and percentages, and PA levels (means—$\bar{\;x}$) and standard deviations—±SD) were calculated using the IPAQ-LF.

In order to verify if analysed variables (LTPA: VPA, MPA, walking) were characterized by a normal distribution, the Kolmogorov–Smirnov test was used for a single sample. Due to a lack of meeting the above assumption for dependent variables (*p* < 0.05), the statistical inference was based on the Mann–Whitney U test. The level of significance was set at α = 0.05.

Relationships between meeting and not meeting the dose of PA required for health recommendations (according to WHO) and analysed socio-demographic criteria were evaluated using the Chi^2^ test. The level of significance is α = 0.05.

Finally, binary logistic regression (method = enter) analyses were carried out in order to determine the influencing factors of meeting the dose of PA required for health recommendations. Sex, chronological age, level of education, place of residence and occupational status were used as independent variables. In a first step, a general model was estimated in which the different years of observation are also included as an independent variable. Next, logistic regression modelling was applied to the different observation years. Nagelkerke R² was used as a measure for goodness of fit. The different models were tested for multicollinearity, outliers and leverage points. No problem with the data could be found with regard to these aspects.

## 3. Results

In the analysed period, 2014–2018, the LTPA of Poles significantly increased (*p* < 0.001). In 2014, the percentage in this scope was 62.4%, in 2015—62.5%, in 2016—61.5%, in 2017—66.6%, and in 2018—70.1%. A growth of participation concerned both men (*p* < 0.001) and women (*p* < 0.001). There were no differences between these groups in this scope, although men were more active. In 2018, their mean weekly energy expenditure for LTPA amounted to 1537.1 ± 2322.8 MET-min/week, while the result for women was 1442.9 ± 2156.5 MET-min/week (Table 2). Since 2016, when a clear growth of LTPA was being noted, the expenditure had increased 1.5 times (in 2016, among men it was 898.9 ± 1443.5, and among women—929.9 ± 1366 MET-min/week).

An analysis of particular types of activities undertaken by Poles in the years 2014–2018 under LTPA showed that men participated significantly more often (*p* < 0.001) than women in VPA (349.2 ± 1087.1 and 260.5 ± 916.1 MET-min/week, respectively) and MPA (283.4 ± 676.2 and 235.3 ± MET-min/week, respectively) (Table 2). On the other hand, women more often walked (*p* < 0.001) (563.9 ± 916.1 and 532.1 ± 917.8 MET-min/week, respectively).

In the years 2014–2018, the percentage of Poles meeting WHO PA recommendations increased (Table 3). In 2016, the recommendations were met by 39.8% men and 44.2% women, while women achieved these more often (*p* < 0.05). In 2018, there were no significant differences between them, but the fraction of active persons in this scope was larger (men—51.4%; women—49.4%). On average, in the years 2014–2018, LTPA at the pro-health level (according to WHO) was realised by 43.9% men and 43.5% women. A Chi^2^ analysis revealed significant differences, depending on age (*p* < 0.001), education (*p* < 0.001), place of residence (*p* < 0.001), and social and occupational status (*p* < 0.001).

A conducted logistic regression revealed that in the years 2014–2018, the probability of realisation of the PA dose recommended by WHO by women was lower than by men (1.1 times). However, 2017 results had a large influence on the above (Table 4). The probability was also conditioned by the age of Poles in the study. In respect of the youngest persons (aged 15–29), people aged 30–39, 40–49 and over 60 had a 1.4 times lower chance to undertake LTPA in the recommended dose, while persons aged 50–59 even 1.6 times lower. Education of Poles was an essential factor having an influence on realisation of pro-health recommendations for PA. They were met relatively more often by respondents with higher and secondary education (*p* < 0.001). A logistic regression revealed that among the less-active groups, persons with basic vocational education were susceptible even to 1.2 times higher risk of not meeting these recommendations in relation to persons with primary education (Table 4). Another factor conditioning undertaking LTPA at a recommended level was the place of residence. Significant differences in this area concerned, in general, all analysed years. The group of Poles who more often met WHO recommendations consisted more often of persons living in towns (1.5 times more in towns <500,000 of inhabitants and nearly 2 times in towns over 500,000 inhabitants) than respondents living in villages. Social and occupational status was also of a great importance. The most active ones, i.e., students/pupils, nearly 2 times more often met these recommendations than white-collar workers (accepted as the reference group). In relation to that group, physical workers were 1.5 times more vulnerable to the risk of a lack of LTPA at a pro-health level, while the risk for farmers was over 2 times higher.

## 4. Discussion

This work presents the first analysis of leisure-time physical activity of Poles aged 15–69 in terms of time trends (2014–2018). A particular focus is paid to analysing the relation of social and demographic factors and probability of meeting pro-health recommendations of the World Health Organisation (WHO), what has a special importance from the perspective of limiting the risk factors of non-communicable diseases (NCDs). A certain advantage of this work is the use of combined data from nationwide cross-sectional studies and a large sample consisting of 7347 persons.

Results show that LTPA of Poles is increasing, what is in line with trends observed in other European countries, such as: Belgium [11], Denmark [29], the Netherlands [30], Sweden [31], Switzerland [32] and United Kingdom [33]. In the period 2016–2018, the percentage of people declaring participation in LTPA increased from 62.4% to 70,1%, and the energy expenditure (being a result of LTPA), over 1.5 times. To compare, in the years 2000–2010, the participation in sport in Spain (although was lower), increased from 46% to 52% among men, and from 27% to 33% among women. It should be also noted that Spanish respondents were older, i.e., aged 15–74 [12].

The presented data indicate that the perception of PA in Poland changed in the course of time. In the past, LTPA was regarded as a unique form of behaviour, while more recently active ways of spending leisure time became acceptable, and even normative behaviour among Poles, especially in the context of taking care of one’s own health (which is indicated by an increasing percentage of people meeting pro-health recommendations by WHO). Research conducted by the Central Statistical Office of Poland shows that the main motive for Poles’ participation in LTPA is pleasure and entertainment (59%), maintaining physical condition and a proper figure (24%). An important factor is also the desire to improve health (11%) [34]. An increasing share of women participating in LTPA is quite visible in Poland. Despite the fact that there are still differences in this scope between men and women (in favour of men), the growth of participation (in the years 2014–2018) does not depend on sex. In the majority of European countries [35,36,37], men more often perform sports than women. However, international studies also prove that since the mid 1990s, women have successively decreased the difference in LTPA participation in relation to men [11,38], what is confirmed also by our results.

Results indicate the stability of forms of spending leisure time undertaken. Regardless the year of study (2014–2018), men significantly more often declared intensive and moderate activities, while women declared walking. The last fact is noted also by Downward and Rasciute [39] and Grima et al. [2] who also add that this has not relation with the leisure time women have at their disposal. However, with no doubt, women (also Polish women [40] due to numerous obligations, taking care of children and beloved ones have, in general, a larger problem of engagement in LTPA [2,41]. It is difficult to state why Polish women prefer walking. Maybe, as it is claimed by Hands et al. [42], undertaking PA (at a particular level, in certain form and of certain intensity) depends on things a respondent wants to achieve. In the case of men, it may be about musculature or looking for challenges [43,44], in the case of women—more about lifestyle factors (social interactions, pleasure [45,46], general activation, slim figure) [45,47,48]—which is indicated by the most modern Polish studies [49]. Ball et al. [50] claim that feeling too fat is not related with the perception of a bad state of health. Thus, this may be a reason why women meet WHO recommendations less often than men. On the one hand, this may reveal the problem of marginalising or scarce knowledge of women about harmful effects of being overweight and obesity. On the other hand, this may suggest that the positive pro-health influence of regular PA is more visible among men. Of course, these deliberations are hypothetical (although not diverging from the most modern Polish evidence) [51]. This fact may be a negation of numerous past reports, according to which the pro-health motive is the factor that increases the frequency of practicing sport, especially among women [46] (more rarely among men) [45].

In the years 2014–2018, WHO recommendations concerning pro-health dose of PA were met, on average, by 43.9% of Polish men and 43.5% of Polish women. This result is quite low in comparison to citizens of Central and Eastern Europe (57.7%) [52]. This is depicted by the direction towards which corrective actions should go. Unfortunately, due to the fact that such study was conducted to such an extensive scope in Poland for the first time, a comparison with other Polish research is not possible. Their character is regional. It is similar in terms of the content, and the study by Macek et al. [53], concerning the region of Kielce (Świętokrzyskie voivodeship), cannot function as a reference as it utilised the authorship classification of levels of PA by Biernat and Tomaszewski [54] instead of IPAQ [28].

The percentage of Poles meeting WHO recommendations has been increasing every year since 2015. The probability of following these recommendations was conditioned by key social and demographic factors, i.e., sex, age, education, place of residence, and social and occupational status. However, it must be mentioned that not all of them modified the behaviour in following years. Such variables as, for example, education of respondents had a significant importance only in the whole analysed period. That means that in the years 2014–2018, Poles with higher and secondary education more often met WHO recommendations than others. On the one hand (looking at the whole period of the study), it confirms the generality of statements that the educational level still determines LTPA [11], i.e., a higher education increases chances of undertaking PA [23,36]. On the other hand (looking at particular years of the study, i.e., 2014, 2015, 2016, 2017, 2018), it is observable that the education of Poles did not modify behaviour of this type during a certain year. Based on their systematic review, Gidlow et al. [55] reported that in 24 studies there was a negative association between PA and educational level, while in 17 studies there was a positive association. However, it must be stressed that in Poland a successive increase of the percentage of persons meeting WHO recommendations in all groups of education level was observed, what is explained by Scheerder and Vos [11] by a global growth of education status (thus, also the awareness of the necessity of undertaking PA).

Another predictor of realisation of a pro-health PA dose is the age of Poles. The greatest opportunity in this scope was for of the youngest group (aged 15–29), which does not raise doubts in the light of previous studies [56]. As a comparison, persons aged 50–59 have the least chance of achieving such a level. Admittedly, modern reports more often show trends of a decrease of LTPA in even older age groups [57,58,59], but the studies by Biernat and Piątkowska [60] show that the problem of inactivity starts at the age of 50 years. This concerns especially those who have no parental responsibilities and can have time for themselves. WHO recommendations are not met by as much as 46.1% of professionally active and 50.3% unemployed Poles aged 50–64 without children aged below 18 [60]. This indicates the necessity of focusing in local corrective programs on this period of life (including, related life events, which can have a significant influence on a change of people’s behaviour). The impact of lifelong physical inactivity, resulting in the world’s number one health problem, can be reversed even during old age with regular exercise and nutrition [61]. As people are living longer, society must be prepared to handle an aging population. As shown by Roberson’s research [62], physical activity can provide many benefits to the elderly, including maintaining/improving health, eliminating the effects of aging, or creating social bonds—which can result in improved quality of life.

Pro-health behaviour of Poles is conditioned also by the place of their residence. The group of those meeting WHO recommendations is composed most often of people living in towns than in villages, whereas differences concern, in general, all analysed years. Such a phenomenon is observed all over the European Union [63], although in the Netherlands, there is observed a more common participation in LTPA of people living in villages [36]. The above results from the fact that Dutch rural areas are characterised by an advantageous social environment (in terms of a higher social and economical status and safer districts) [64] and physical environment (climate, presence of natural elements and space for practicing sport, etc.) [36].

European evidence suggests that social and occupational status is positively related to participation in LTPA [11,22]. As far as our study is concerned, the most active respondents were students/pupils, who nearly 2 times more often than white-collar workers achieved WHO recommendations by means of LTPA. In relation to that group, manual workers were 1.5 times more vulnerable to the risk of a lack of LTPA at a pro-health level, while the risk for farmers was over 2 times higher. Due to compulsory physical education classes, this result is not surprising. It is also not surprising that Poles who are non-manual workers more often (despite time limitations increasing along with promotions; Hallmann and Breuer [17]) follow pro-health recommendations. Once again, it must be emphasised that Polish farmers are vulnerable to a great risk of having NCDs [65,66].

Our analysis shows that a breakthrough year for the whole group of Poles under this study was 2016, in which chances of realisation of the pro-health recommendations of the WHO increased significantly (in comparison to 2014), although the observed differences were not significant. However, in 2017 the chances were significantly higher by 1.4 times, and in 2018—by 1.5 times, which indicates a trend in the behaviour of Poles. This may be a result of the Rodzina 500 plus program (Family 500+) [67] introduced on 1 April 2016 aimed at supporting families in upbringing their children in the form of monthly child-support benefits for each child in a family in the amount of PLN 500. However, it should be mentioned that this did not concern highly educated persons. In case of respondents with basic vocational and secondary education, the growth of LTPA lasted just till 2017.

## 5. Limitations

A limitation of this study is a subjective (although necessary due to population studies) evaluation of LTPA. It should be also considered that an increasing energy expenditure related with LTPA can be a result of (undetectable on the basis of data) the greater activity of active persons in the initial stage of the study. A self-reported study might also underestimate or overestimate patterns of PA due to recall or social desirability bias. Furthermore, longitudinal surveys may be accompanied by problems, or model limitations, as evidenced by some of the diagnoses presented. In the models of logistic regression, Nagelkerke R^2^ values are low. This suggests that the analysed phenomenon is explained by the utilised model to a small extent.

## 6. Conclusions

The participation of Poles in LTPA has constantly increased and the percentage of people achieving the level of PA recommended for good health is growing, which indicates a trend of behaviour of the citizens of this country. Despite this fact, the PA level recommended by the WHO is met by just over 40% of people—which is definitely lower in comparison to other citizens of Central and Eastern Europe (57.7%). Therefore, it is necessary not only to continue campaigns aiming at increasing the PA level of Poles, but also to intensify these activities. The results of this study show that participation in LTPA at a health maintenance level determines a broader model of variables (i.e., age, gender, place of residence, professional status) than those included in WHO recommendations. It seems reasonable to develop Polish recommendations and guidelines for specific groups, including females and males, those who live in the villages and in the towns, different age groups: children/adolescents, adults (aged 50 to 59—most vulnerable to physical inactivity) the elderly, and various professional groups (especially farmers and physical workers).

## Figures and Tables

**Table 1:** Characteristics of the samples in 2014–2018.

Factors		Year of the Survey					
		2014 (*n* = 1019)	2015 (*n* = 1020)	2016 (*n* = 2118)	2017 (*n* = 2131)	2018 (*n* = 1059)	Total (*n* = 7347)
Sex	Male (*n* = 3551)	48.3	48.4	48.2	48.5	48.3	48.3
	Female (*n* = 3796)	51.7	51.6	51.8	51.5	51.7	51.7
Age	15–29 years (*n* = 1628)	22.5	22.5	22.8	21.7	21.3	22.2
	30–39 years (*n* = 1425)	19.2	19.0	19.5	19.5	19.4	19.4
	40–49 years (*n* = 1115)	14.9	14.6	14.9	15.4	16.0	15.2
	50–59 years (*n* = 1183)	16.8	16.8	16.2	15.9	15.0	16.1
	60–69 years (*n* = 1996)	26.6	27.1	26.6	27.5	28.3	27.2
Level of education	Primary (*n* = 1259)	18.9	16.3	15.5	17.9	17.9	17.1
	Basic vocational (*n* = 2308)	31.2	32.9	30.8	31.1	31.9	31.4
	Secondary (*n* = 2724)	35.8	35.0	36.7	39.0	37.1	37.1
	Higher (*n* = 1056)	14.0	15.8	17.0	11.9	14.4	14.4
Place of residence	Village (*n* = 2848)	38.3	38.8	38.9	38.8	38.9	38.8
	Towns up to 500,000 inhabitants (*n* = 3649)	50.6	50.0	49.3	49.6	49.3	49.7
	Towns with 500,000 inhabitants (*n* = 850)	11.1	11.2	11.8	11.7	11.8	11.6
Occupational status	White-collar workers (*n* = 1925)	21.5	23.2	27.9	26.7	29.1	26.2
	Physical workers (*n* = 1952)	27.4	28.8	24.6	26.4	27.8	26.6
	Farmers (*n* = 271)	7.5	7.0	2.6	2.3	1.7	3.7
	Housewives (*n* = 329)	3.6	3.1	5.0	5.4	3.7	4.5
	Retirees/pensioners (*n* = 1865)	23.0	23.9	25.8	26.6	25.8	25.4
	Students (*n* = 557)	7.9	6.7	8.0	7.4	7.7	7.6
	Unemployed (*n* = 448)	9.2	7.3	6.0	5.0	4.2	6.1

**Table 2:** LTPA (VPA, MPA, Walking) in MET-min/week declared by males and females in 2014–2018 ($\bar{\;x}$ and ±SD).

LTPA	2014–2018	2014	2015	2016	2017	2018
VPA						
Males (*n* = 3551)	349.2 ± 1087.1 ***	353.7 ± 1096.4 *	253.5 ± 872.4	313.6 ± 940.7	374.6 ± 1168.1 **	457.7 ± 1340.0
Females (*n* = 3796)	260.5 ± 916.1	215.3 ± 860.9	258.5 ± 905.2	257.9 ± 779.6	239.5 ± 947.6	354.9 ± 1140.5
Total (*n* = 7347)	303.4 ± 1003.8	282.2 ± 983.6	256.1 ± 889.0	284.7 ± 861.2	305.0 ± 1062.2	404.7 ± 1241.6
MPA						
Males (*n* = 3551)	283.4 ± 676.2 ***	268.7 ± 599.1 ***	190.4 ± 494.8	384.3 ± 710.0	259.9 ± 747.2 **	232.9 ± 655.6
Females (*n* = 3796)	235.7 ± 608.0	159.5 ± 437.3	158.2 ± 447.3	392.1 ± 726.6	166.7 ± 575.5	207.6 ± 629.4
Total (*n* = 7347)	258.7 ± 642.3	212.2 ± 524.3	173.8 ± 470.9	388.3 ± 718.5	211.9 ± 665.8	219.8 ± 642.0
Walking						
Males (*n* = 3551)	532.1 ± 917.8 **	417.0 ± 792.2 *	384.9 ± 721.3 **	201.0 ± 501.2 **	830.3 ± 1095.8	846.5 ± 1138.4
Females (*n* = 3796)	563.9 ± 916.1	466.9 ± 763.947	477.7 ± 752.0	280.0 ± 589.4	780.7 ± 1063.6	880.5 ± 1180.4
Total (*n* = 7347)	548.6 ± 917.0	442.8 ± 777.7	432.7 ± 738.4	242.0 ± 550.0	804.7 ± 1079.4	864.0 ± 1159.8
Total						
Males (*n* = 3551)	1164.6 ± 1910.4	1039.4 ± 1750.5	828.8 ± 1377.1	898.9 ± 1443.5	1464.8 ± 2276.6	1537.1 ± 2322.8
Females (*n* = 3796)	1060.1 ± 1682.6	841.7 ± 1377.0	894.3 ± 1468.2	929.9 ± 1366.0	1186.9 ± 1882.9	1442.9 ± 2156.5
Total (*n* = 7347)	1110.7 ± 1797.0	937.1 ± 1570.8	862.6 ± 1424.5	915.0 ± 1403.6	1321.7 ± 2087.3	1488.5 ± 2238.0

Abbreviations: $\bar{\;x}$ mean; ±SD: standard deviation; LTPA: leisure-time physical activity; VPA: vigorous physical activity; MPA: moderate physical activity; *p*: level of significance; * *p* < 0.05; ** *p* < 0.01; *** *p* < 0.001.

**Table 3:** Percentage of adult Poles meeting WHO PA recommendations in 2014–2018.

Factors	2014–2018	2014	2015	2016	2017	2018
Sex						
Males (*n* = 3551)	43.9	40.7	37.0	39.8	49.3	51.4
Females (*n* = 3796)	43.5	38.5	37.1	44.2	45.5	49.4
*p*	NS	NS	NS	<0.05	NS	NS
Age						
15–29 years (*n* = 1628)	55.7	62.9	50.7	49.0	56.7	65.9
30–39 years (*n* = 1425)	42.7	40.3	38.1	39.6	46.5	48.3
40–49 years (*n* = 1115)	40.8	27.6	38.9	39.0	44.4	50.6
50–59 years (*n* = 1183)	36.9	30.4	27.5	40.8	40.4	38.2
60–69 years (*n* = 1996)	40.3	31.7	29.7	40.4	46.2	46.3
*p*	<0.001	<0.001	<0.001	<0.05	<0.001	<0.001
Level of education						
Primary (*n* = 1259)	47.3	41.5	39.8	45.7	50.8	55.6
Basic vocational (*n* = 2308)	35.7	31.1	26.5	33.8	41.2	42.2
Secondary (*n* = 2724)	46.6	40.5	40.6	43.8	51.8	52.3
Higher (*n* = 1056)	49.5	53.1	48.4	50.0	43.3	57.4
*p*	<0.001	<0.001	<0.001	<0.001	<0.001	<0.01
Place of residence						
Villages (*n* = 2848)	37.7	36.2	28.0	35.7	43.3	41.1
Towns up to 500 thous inhabitants (*n* = 3649)	46.0	39.7	41.4	44.4	48.5	54.9
Towns with 500 thous inhabitants (*n* = 850)	54.3	50.4	49.1	53.4	55.8	61.7
*p*	<0.001	<0.05	<0.001	<0.001	<0.01	<0.001
Occupational status						
White-collar workers (*n* = 1925)	50.2	51.1	45.1	48.4	50.0	57.1
Physical workers (*n* = 1952)	36.2	29.0	32.0	30.5	42.8	45.2
Farmers (*n* = 271)	22.2	18.4	15.5	26.8	32.7	22.2
Housewives (*n* = 329)	45.9	37.8	28.1	50.5	51.7	38.5
Retirees/pensioners (*n* = 1865)	40.4	34.6	33.2	41.9	43.0	43.7
Students (*n* = 557)	67.5	82.5	63.2	55.6	68.4	79.3
Unemployed (*n* = 448)	44.2	37.2	44.6	43.0	50.5	46.7
*p*	<0.001	<0.001	<0.001	<0.001	<0.001	<0.001
Total (*n* = 7347) (*p* < 0.001)	43.7	39.5	37.1	42.1	47.3	50.3

Abbreviations: PA: physical activity; WHO: World Health Organization; *p*: level of significance; NS: non-significant.

**Table 4:** Binary logistic regression for meeting WHO recommendations in physical activity among Polish society 2014–2018, in odds ratios [Exp(β)].

Variables	2014–2018	2014	2015	2016	2017	2018
Constant	0.847	1.38	0.56	0.84	1.11	1.23
Sex						
Males (*n* = 3551) (ref)						
Females (*n* = 3796)	0.9 *	0.89	0.98	1.02	0.79 *	0.89
Age						
15–29 years (*n* = 1628) (ref)						
30–39 years (*n* = 1425)	0.73 ***	0.59 *	0.8	0.72 *	0.85	0.65
40–49 years (*n* = 1115)	0.72 ***	0.36 ***	0.89	0.79	0.83	0.75
50–59 years (*n* = 1183)	0.63 ***	0.42 **	0.5 **	0.86	0.72	0.46 **
60–69 years (*n* = 1996)	0.7 ***	0.42 **	0.49 *	0.69	1.04	0.76
Level of education						
Primary (*n* = 1259) (ref)						
Basic vocational (*n* = 2308)	0.84 *	1.11	0.72	0.76	0.86	0.94
Secondary (*n* = 2724)	1.05	1.18	1.12	0.96	1.13	1.06
Higher (*n* = 1056)	1.07	1.53	1.36	1.1	0.73	1.09
Place of residence						
Villages (*n* = 2848) (ref)						
Towns up to 500,000 inhabitants (*n* = 3649)	1.41 ***	1.07	1.73 ***	1.44 ***	1.25 *	1.93 ***
Towns with 500,000 inhabitants (*n* = 850)	1.83 ***	1.44	2.07 **	1.88 ***	1.66 **	2.22 ***
Occupational status						
White-collar workers (*n* = 1925) (ref)						
Physical workers (*n* = 1952)	0.66 ***	0.46 **	0.82	0.58 ***	0.74 *	0.65 *
Farmers (*n* = 271)	0.48 ***	0.38 *	0.63	0.59	0.59	0.39
Housewives (*n* = 329)	1.03	0.69	0.73	1.33	1.26	0.51
Retirees/pensioners (*n* = 1865)	0.85	0.78	1.28	1.03	0.7 *	0.6
Students (*n* = 557)	1.79 ***	3.2 **	2.22 *	1.2	1.85 *	2.47 *
Unemployed (*n* = 448)	0.97	0.65	1.57	0.97	1.07	0.78
Year of survey						
2014 (*n* = 1019) (ref)						
2015 (*n* = 1020)	0.91					
2016 (*n* = 2118)	1.07					
2017 (*n* = 2131)	1.36 ***					
2018 (*n* = 1059)	1.54 ***					
*n*	7347	1019	1020	2118	2131	1059
Nagelkerke. R^2^	0.08	0.17	0.11	0.06	0.05	0.11

Abbreviations: ref, reference group; p, level of significance; * *p* < 0.05; ** *p* < 0.01, *** *p* < 0.001.
